# Caregivers’ Perspectives on Changes in Family Life During B-ALL Therapy: A Qualitative Study From the Children’s Oncology Group

**DOI:** 10.1002/pbc.32057

**Published:** 2025-09-19

**Authors:** Alexandra Dreyzin, Megan Ware, Katrina Stumbras, John Kairalla, Emily Hibbitts, Brenna Mossman, Lyn Balsamo, Rozalyn Rodwin, Kirsten K. Ness, Claire C. Conley, Elizabeth Raetz, Meenakshi Devidas, Lillian Sung, Mignon Loh, Stephen P. Hunger, Reuven J. Schore, Anne Angiolillo, Nina Kadan-Lottick

**Affiliations:** 1 Pediatric Oncology Branch and Center for Cellular Engineering, National Institutes of Health Clinical Center, Bethesda, Maryland, USA; 2 Department of Kinesiology, Health Promotion, and Recreation, University of North Texas, Denton, Texas, USA; 3 Department of Pediatrics, Children’s National Hospital, Washington, District of Columbia, USA; 4 Department of Biostatistics, Colleges of Medicine and Public Health & Health Professions, University of Florida, Gainesville, Florida, USA; 5 Lombardi Comprehensive Cancer Center, Georgetown University, Washington, District of Columbia, USA; 6 Department of Pediatrics, Yale University School of Medicine, New Haven, Connecticut, USA; 7 Department of Epidemiology and Cancer Control, St. Jude Children’s Research Hospital, Memphis, Tennessee, USA; 8 Perlmutter Cancer Center and Department of Pediatrics, NYU Langone Medical Center, New York, New York, USA; 9 Department of Global Pediatric Medicine, St. Jude Children’s Research Hospital, Memphis, Tennessee, USA; 10 Department of Paediatrics, The Hospital for Sick Children, Toronto, Ontario, Canada; 11 Ben Towne Center for Childhood Cancer and Blood Disorders Research, Seattle Children’s Research Institute and the Department of Pediatrics, Seattle Children’s Hospital, Fred Hutch Cancer Center, University of Washington, Seattle, Washington, USA; 12 Department of Pediatrics and the Center for Childhood Cancer Research, Children’s Hospital of Philadelphia and the Perelman School of Medicine at the University of Pennsylvania, Philadelphia, Pennsylvania, USA; 13 Division of Oncology, Center for Cancer and Blood Disorders, Children’s National Hospital, Washington, District of Columbia, USA; 14 Department of Pediatrics and Clinical and Translational Oncology Program, George Washington University School of Medicine and Health Sciences, Washington, District of Columbia, USA; 15 Servier Pharmaceuticals, Boston, Massachusetts, USA

**Keywords:** family life, financial, leukemia, parent perspective, quality of life, school, work

## Abstract

**Background:**

Treatment of pediatric B-acute lymphoblastic leukemia (B-ALL) impacts both patients and their caregivers. An understanding of family functioning during therapy can inform family-centered care. We aimed to prospectively identify negative and positive changes in family life as perceived by caregivers throughout ALL therapy.

**Methods:**

Caregivers of children aged ≥4 years with average-risk B-ALL enrolled on the Children’s Oncology Group trial AALL0932 who consented to an ancillary study were asked: “How has family life changed since your child’s diagnosis of leukemia for the better or for the worse?” Written free responses were collected at approximately 2, 8, 17, 26 (end of therapy for females), and 38 (end of therapy for males) months post-diagnosis. Inductive content analysis was used to create codes, subcategories, and categories. Descriptive statistics were used to characterize the sample and frequencies of reported codes.

**Results:**

Overall, 994 responses were collected from caregivers of 468 children across all timepoints. Twenty-seven individual codes were identified, categorized by negative changes (reported by 89% of caregivers) and positive changes (reported by 58% of caregivers). Subcategories of negative changes, including changes in daily routines, work and finance, patient health and care needs, effects on other family members, and emotional changes, were identified across all timepoints, but were most prevalent early in therapy. Importantly, positive changes were also identified, including family support, community support, and changes in outlook.

**Conclusion:**

This study identifies negative and positive family changes perceived by caregivers of children undergoing B-ALL therapy that can inform future interventions to better support families.

## 1 ∣ Introduction

Therapy for pediatric acute lymphoblastic leukemia (ALL), the most common childhood cancer, has resulted in excellent overall 5-year survival outcomes of greater than 90% [1]. However, the treatment course lasts over 2 years and has important consequences for patients as well as their families [2, 3]. Patients experience toxicities that require caregiving support, including behavior changes, peripheral neuropathy, nausea, and fatigue [3-6]. Families must keep up with multiple medical appointments, unscheduled emergency department visits, and hospitalizations that disrupt daily life [7, 8].

The effect of ALL therapy on children’s social, emotional, and physical functioning has been documented using quality-of-life survey data from large Children’s Oncology Group (COG) trials [9, 10]. Although most quality-of-life studies focus on the patient, chronic illness has a wide-ranging impact on the quality of life for patients’ family members [11, 12]. A survey completed in conjunction with a recent ALL clinical trial reported the incidence of disruptive family events such as employment changes, divorce, or relocation, but did not capture caregivers’ perspectives on how these events affected them [13].

Several qualitative studies have examined topics related to ALL therapy through in-depth interviews with small groups of caregivers (primarily parents). These studies focused on specific topics selected by the research teams, such as the decision to enroll in clinical trials [14], lifestyle changes related to oral chemotherapy [15], and decision-making surrounding returning to school [16]. Broad insights from an open-ended survey of caregivers may reveal additional important family experiences that can inform providers and allow for more effective, family-centered support.

In this study, we examined free-text responses from caregivers (largely parents) whose children with standard-risk B-ALL were enrolled on the COG study AALL0932 (NCT01190930) [3]. A total of 468 caregivers provided free-text responses across five timepoints, indicating how family life had changed since their child’s diagnosis. The aim of this study was to identify codes and categories among caregivers’ responses using inductive content analysis in order to inform potential future interventions.

## 2 ∣ Methods

The primary Children’s Oncology Group (COG) clinical trial, AALL0932 (NCT 01190930), has been described in detail elsewhere [3]. In brief, children with National Cancer Institute (NCI) standard-risk B-cell ALL were enrolled from 2010 to 2018 on this Phase III randomized, multicenter trial. Females were treated for 2 years and males for 3 years from the start of Interim Maintenance I. Participants enrolled on COG AALL0932 who were ≥4 years old and had English-speaking parents were eligible for a longitudinal study of quality-of-life outcomes. This survey, developed by a multidisciplinary team of pediatric oncology providers, included the open-ended question pertaining to family functioning post-diagnosis. As patients were consented for the larger therapeutic study, they were also offered the opportunity to participate in the ancillary study until the accrual goal of 600 patients was met. The institutional review boards of all participating sites approved this study in accordance with the ethical standards of the Helsinki Declaration. Informed consent was obtained from all participants.

### 2.1 ∣ Data Collection

As part of the longitudinal quality-of-life study, caregivers were asked to respond to the open-ended, free-text prompt: “How has family life changed since your child’s diagnosis of leukemia for the better or for the worse?” Responses were provided on paper at the time of clinic visits. This question was asked of caregivers at five timepoints throughout therapy: Timepoint 1 at 2 months (end-consolidation), Timepoint 2 at 8 months (start of maintenance), Timepoint 3 at 17 months (maintenance month 10), Timepoint 4 at 26 months (end of therapy for females, Maintenance month 18 for males), and Timepoint 5 at 38 months (end of therapy for males).

### 2.2 ∣ Qualitative Analysis

Inductive content analysis was used to analyze the free-response data [17, 18]. Transcribed responses were provided to researchers in random order without any identifying information or additional demographics, and each respondent was assigned a number. Quotations included below are labeled as “R#T#,” denoting this randomly assigned respondent number and timepoint. Iterative coding and readings (five total iterations) were used to develop codes for the free-response data. A final codebook with definitions of each code was created for use in the process to ensure consistency among coders. This study was driven by an interpretivist paradigm, in which we sought to understand the lived experiences of caregivers through examination and interpretation of their written responses [19, 20]. Two independent investigators (A.D., K.S.) coded all the responses for consensus. During the time of this study, A.D. was a pediatric hematology/oncology fellow with prior experience in qualitative analysis, and K.S. was a pediatric resident. Any discrepancies between coders were resolved with a third investigator (R.R.). Coders remained reflexive throughout the coding process. Subcategories were created by grouping the final codes by similar context across responses, and subcategories were grouped to create overarching categories (Supporting Information).

### 2.3 ∣ Quantitative Analysis

Demographics were reported using descriptive statistics, and the group of respondents to the free-response question was compared to the overall survey respondents using chi-square tests. Content analysis was used to provide an overview of code frequencies [21, 22]. The number of caregivers who mentioned each code, as well as the number that mentioned any negative or positive change, was tabulated. For this calculation, if a code was mentioned by a caregiver at multiple timepoints, it was counted only once. Frequencies of responses that mentioned negative and positive changes were tabulated for each timepoint. The proportion of responses that included negative and positive changes was tabulated for T1 and T4 (the last timepoint, inclusive of both males and females). For those who had paired responses available at both T1 and T4, McNemar’s test was used to compare proportions of negative and positive changes between these timepoints. A *p*-value of less than 0.05 was considered significant, and calculations were performed using GraphPad Prism Version 10.1.

## 3 ∣ Results

### 3.1 ∣ Participants

A total of 600 participants enrolled in the quality-of-life study. Subsequently, six were deemed ineligible, and 39 did not complete any evaluations. Among 555 participants who consented, were eligible, and submitted survey data, 468 completed the free-response question for at least one timepoint. Participant demographics are summarized in Table 1: median age was 5.6 years, 46% were female, and 69% identified as non-Hispanic White. Demographics did not differ significantly between patients whose caregivers did and did not respond to the free-response survey question. Across timepoints, there were a total of 994 responses as follows: 360 responses at Timepoint 1, 219 at Timepoint 2, 182 at Timepoint 3, 169 at Timepoint 4, and 64 at Timepoint 5. Responses to the free-response question as a proportion of total survey respondents are shown in Table 2.

### 3.2 ∣ Identification of Themes

**FIGURE 1 ∣:**
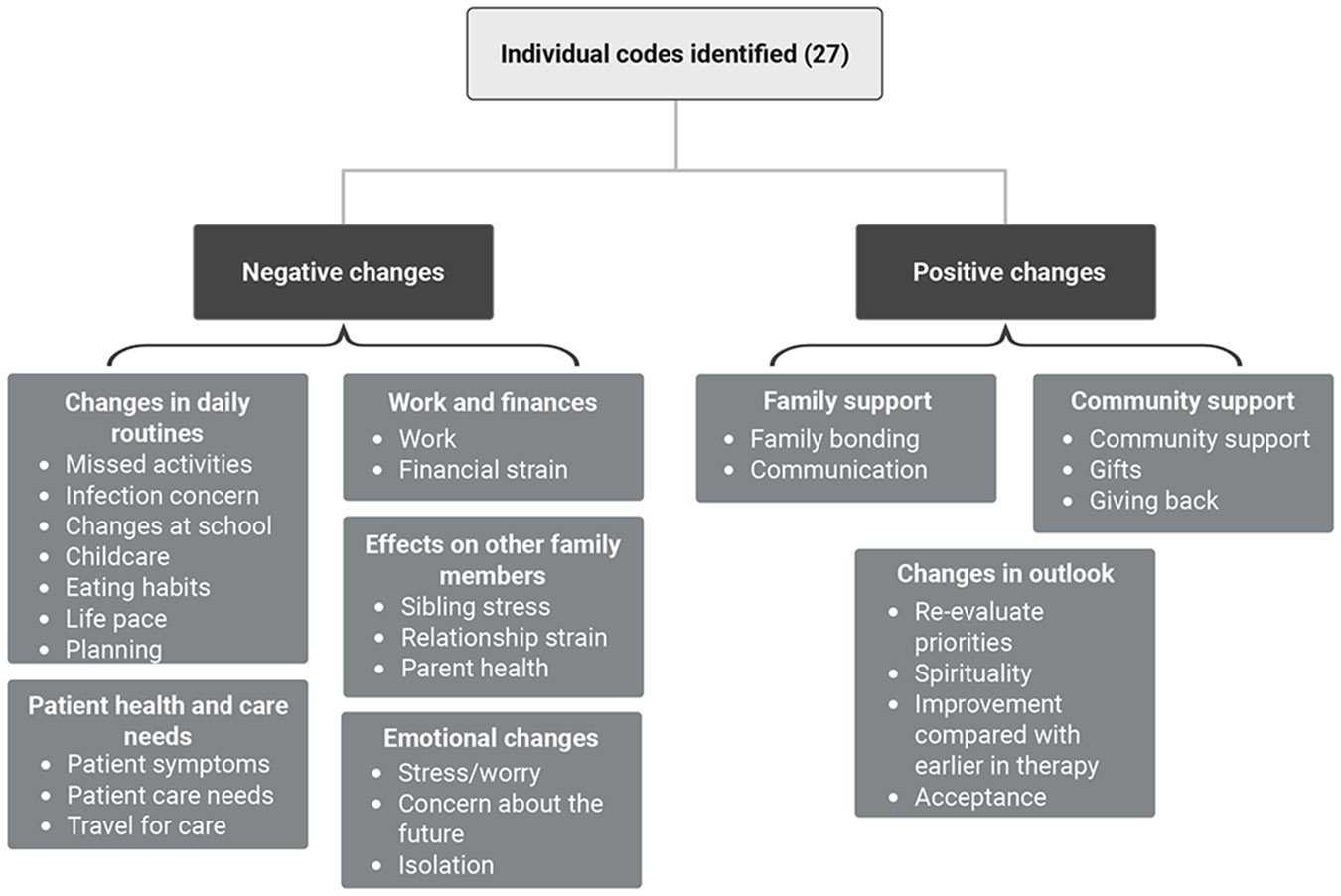
Codes organized into categories and subcategories.

Twenty-seven individual codes were identified (Table 3; denoted by italicized font). They were further categorized into Negative Changes, comprised of five subcategories, and Positive Changes, comprised of three subcategories (Figure 1).

#### 3.2.1 ∣ Negative Changes

##### 3.2.1.1 ∣ Changes in Daily Routines.

Many of the responses described negative changes to the daily life of both patients and families. *Missed activities* were commonly mentioned, with families limiting vacations, social gatherings, church, and extracurricular activities. One response described “Increased overall sadness by myself and my husband that our daughter misses out on play and other opportunities.” (R407T1). For many, the missed activities were directly linked to the code of *infection concern*, with caregivers explaining that they “Did not put her into swimming classes because of fear of infection” (R369T4) or “He has felt left out at times from family functions due to infection precautions.” (R191T3).

Caregivers also worried about *changes at school*, including missed schoolwork and changes to homeschooling schedules. Some had to “spend more time working with [patient] in the evening on school work, to catch up on work he has missed.” (R85T2). Arranging for *childcare* was also a challenge, whether caring for the sick child at home or caring for siblings during appointments.

Another identified code was a change in *eating habits* for both the patient and family members. Some families made an effort to eat healthier, while others ate fast food more frequently for convenience. Caregivers described thinking more about both timing and content of meals, as “dinnertime is strictly scheduled due to chemo,” (R243T2) and “patient’s taste and appetite is all over the place.” (R229T2).

Perceptions of *life pace* also changed, with caregivers feeling that “life is more hectic” (R133T1) and expressing “difficulty balancing career and family.” (R186T1). Similarly, caregivers expressed *difficulty planning*, noting an inability to make long-term plans: “All plans are up in the air, we live day by day, one minute you’re home, the next we’re in the hospital.” (R151T1).

##### 3.2.1.2 ∣ Work and Finances.

For caregivers, *changes at work* were common, with many references to quitting jobs, reducing work hours, working from home, or working extra hours. Furthermore, some reported that the leukemia diagnosis was “affecting focus and performance at work.” (R161T1). *Financial strain* was a closely linked code, often described in association with lost income. For example, one parent explained: “My husband is losing about $1200 a month in overtime pay so that he can take time off work when we have chemotherapy.” (R205T1). Another wrote that even though they “cut back on all extras, cut off home phone and cable The financial worries are always there.” (R107T3). For some caregivers, changes in food preferences, as described above, were also linked with financial strain: “Grocery bill has gone up on steroids,” (R221T1) and another stated that “[the patient] don’t like eating what others are eating so we end up ordering out for him. That costs a lot of money.” (R392T4).

##### 3.2.1.3 ∣ Patient Health and Care Needs.

Concern about *patients’ symptoms* referred to symptoms like hair loss, nausea, fatigue, weight changes, and pain. Behavioral changes were also described, including anxiety, anger, concentration difficulties, sleep disturbances, or worries about body image. One caregiver wrote, “I hate when people look at my daughter…. She thinks she is a male because all of her hair has fallen out.” (R330T1). Some responses attributed symptoms to chemotherapy or specifically to steroids: “Dex[amethasone] creates tension between parents; 2–4 h/day of non-stop shrieking, screaming, crying, throwing objects, yelling at parents, pure rage and despair each day for 5 days. Enough to break a parent’s heart and break the family apart. Unbelievable difficult burden of therapy.” (R172T3). Additionally, caregivers described the burden of *patient care needs*, including “daily medications and scheduled visits for procedures and check-ups or blood transfusions.” (R374T4). For some, the need to *travel for care* was also significant, requiring them to “purchase another vehicle” (R183T2) or even “live away from home for treatment.” (R75T1).

##### 3.2.1.4 ∣ Effects on Family Members.

Caregivers described how leukemia therapy impacted other family members, with *sibling stress* being common. For example, one stated, “I have not been home very much for my other two children. I believe my other two kids are struggling. My second child is failing school.” (R211T1). *Relationship strain* between parents was also described, with tension exacerbated by limited time to spend together as a couple, as well as “stress in the family that is handled very differently by each family member.” (R258T2). In addition, *parent health* was affected, as respondents described little time for sleep, exercise, self-care, or their own medical appointments. Some caregivers struggled with their own mental health: “Mother has become very anxious as well as father... Both started anxiety medication.” (R464T3). There were occasional references to a caregiver who “has started using drugs” (R395T4) or whose “alcohol abuse has worsened, causing more family strife.” (R406T3).

##### 3.2.1.5 ∣ Emotional Changes.

Responses included statements of generalized *stress/worry* as well as more specific expressions of *concern about the future*: “Oh, and the fear, the fear, is almost paralyzing at times” (R202T3), one wrote. Caregivers also described feelings of *isolation*, both practically when they “don’t have extra help and hands” (R412T1) and socially, when they are “isolated from family and friends, due to needing to safeguard our son’s health.” (R411T1).

#### 3.2.2 ∣ Positive Changes

##### 3.2.2.1 ∣ Family Support.

Caregivers also described positive changes associated with leukemia treatment. One wrote, “It is hard to believe that your life could change for the better after you find out your 9-year-old son has leukemia, but it is true. We are closer and don’t take anything for granted anymore. You find out just how strong you and your children really are.” (R29T1). Specifically, f*amily bonding* was a common positive change, with many writing that leukemia “has brought everyone closer together for the sake of the child.” (R2T4). Patients and caregivers developed better *communication*, becoming “more assertive and able to articulate needs” (R248T4) and sharing feeling with one another: “[My daughter] and I have shared a lot more fears than we used to. Both are strong females but express when we are scared and reassure each other that its [sic] ok to be scared.” (R203T1).

##### 3.2.2.2 ∣ Community Support.

*Community support* was also identified, with “great support through the school system, friends, coworkers, and community.” (R405T2). Additionally, some described *gifts* as a result of their child’s leukemia diagnosis—being “showered with gifts from friends and family” (R219T1) or “more opportunities to do activities [they] otherwise would not be able to do” (R452T4) through programs like Make- A-Wish. On the other hand, caregivers also appreciated *giving back* to the community, becoming “more involved with creating awareness and fundraising for childhood cancer.” (R408T4).

##### 3.2.2.3 ∣ Changes in Outlook.

Additional positive changes were related to changes in the caregivers’ outlook. For example, the code *re-evaluate priorities* refers to caregivers who wrote that they “quickly realized what was truly important in life,” (R202T3) or developed an “understanding of true priorities, weeding out fair-weather friends, clear focus on financial priorities.” (R143T3). Some found strength in *spirituality*, writing, “We have found comfort in our Faith.” (R150T1). A few noted *improvement* compared with prior timepoints in therapy: “Since being on Maintenance, things have returned to normal.” (R110T3). Others described gaining *acceptance* of the diagnosis: “I felt sad, mad, confused, lost, blank, scared. Nothing was good out of the way I felt. I felt as if my life paused for a minute and I had to think twice about what had just happened. As time went on and days turned into weeks, I’ve learned to accept it and take it one step at a time. I’ve become stronger, wiser, and thank God that my son is still with me.” (R362T1).

### 3.3 ∣ Frequencies of Codes

Overall, 89% of caregivers reported at least one negative change, and 58% reported at least one positive change (Table 4). Respondents each described a median of three negative changes and one positive change. Negative changes that were mentioned by more than 20% of caregivers included *missed activities, sibling stress, financial strain, patient symptoms, changes at work*, and generalized *stress/worry*. Positive changes mentioned by more than 20% of caregivers included *family bonding* and *re-evaluating priorities* (Table 4).

**FIGURE 2 ∣:**
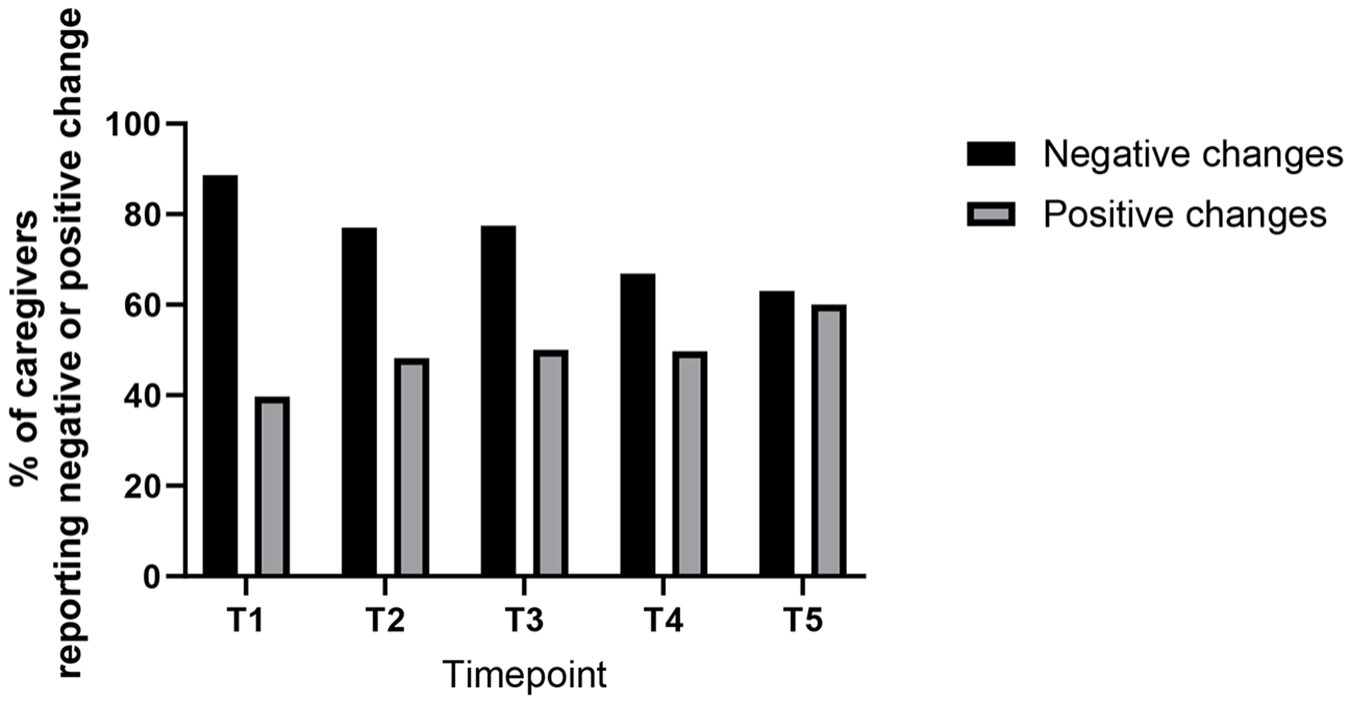
Proportion of respondents reporting negative or positive changes at each timepoint. *T5 only included males. Codes are not mutually exclusive, that is, a caregiver could report both negative and positive changes at the same timepoint.

The proportion of caregivers at Timepoint 4 who reported negative changes was 67%, lower than 89% at Timepoint 1. Accordingly, the proportion of caregivers reporting positive changes was slightly higher at Timepoint 4 (50%) than at Timepoint 1 (40%) (Figure 2). Paired responses at both Timepoints 1 and 4 were available from 123 caregivers. Among these, 108 (89%) reported negative changes at Timepoint 1 and 86 (70%) at Timepoint 4 (*p* = 0.0009). Positive changes were reported by 52 (42%) at Timepoint 1 and 62 (50%) at Timepoint 4 (*p* = 0.16).

As attrition was observed between T1 and subsequent timepoints, those caregivers who had only a T1 response (*n* = 147) were also evaluated separately. Per timepoint, these respondents described the same median number of negative (2) and positive (0) changes as those who had responses at later timepoints. All codes were mentioned at least once in this subgroup, and there was no clear discrepancy in codes in this group that points to a reason for attrition.

## 4 ∣ Discussion

In this multi-site, longitudinal study, 468 caregivers of children with B-ALL provided free-text responses over the 2–3-year timeframe of therapy, sharing how their families’ lives were affected by leukemia diagnosis and therapy. Reported negative changes were subcategorized into changes to daily routines, changes to caregivers’ work and financial situation, concerns about ongoing symptoms, stress on siblings and other family members, and generalized worry. Subcategories of positive changes included family support, community support, and changes in caregivers’ outlook. While many of the themes that families expressed are consistent with prior literature, the persistence of these concerns, not only early in diagnosis but also later into maintenance therapy, is a key finding.

*Missed activities* were the most commonly mentioned negative change to family life. While returning to school after a cancer diagnosis is generally encouraged and guidance has been published, the decision-making surrounding other activities, such as extracurriculars or social events, is left up to individual caregivers [23, 24]. Discussion of return to such activities may help families weigh safety concerns and address barriers to participation. Of note, the surveys reported here took place prior to the COVID pandemic, so public perceptions of infection risk as well as opportunities for remote participation in activities may now differ.

*Financial strain* and *changes at work* were also prominent. Financial strain is a known concern for caregivers of children with cancer, and is a key priority in the WHO Global Initiative for Childhood Cancer [25]. Parents’ job changes during cancer treatment have been associated with greater perceived financial burden across a range of household incomes [26]. Intervention in this area is complex, with limited resources at treatment facilities; however, it is increasingly a focal point in pediatric oncology research, with interventions including financial counseling, navigation services [27], or direct resources like groceries and transportation [28-30]. Our findings support the need for further research aimed at minimizing financial toxicity during leukemia therapy.

Concerns about ongoing symptoms also persisted across timepoints, consistent with prior studies that report symptom burden throughout B-ALL therapy [31]. Higher symptom burden is associated with worse quality of life for patients [32], and caregivers’ perception of symptoms aligns with patients’ perception [33]. Importantly, caregivers have expressed that despite the prevalence of symptoms, normalization of these symptoms by healthcare providers was distressing, as it discouraged hope that symptoms would be resolved [34, 35]. Taken together, these data emphasize the need for frequent symptom assessment and intervention even during later stages of therapy. Electronic patient (or proxy)-reported outcomes, used previously during active chemotherapy or palliative care, may also have a role throughout B-ALL maintenance therapy [36, 37].

Finally, *sibling stress* was identified by about one-third of caregivers. The disruption in family routines as well as the increased attention that patients require from caregivers, can impact sibling well-being. Siblings of childhood cancer patients report post-traumatic stress and describe a need for coping strategies as well as improved communication with healthcare providers [38-41]. Additionally, our data suggest that consideration of siblings may contribute to caregivers’ stress. Support groups specifically targeting siblings, which can reduce siblings’ anxiety and depression [42-44], may also provide relief to caregivers [45].

Although negative changes were more frequently described, positive changes, such as increased family bonding and receiving community support, offer an important perspective, which is not commonly emphasized. It is encouraging that there was a trend of decreased negative and increased positive changes over the course of leukemia therapy, although participation bias is a possibility. Similar trends have been previously reported: for instance, children with cancer described the experience of illness in a more positive way at later timepoints in therapy [46]. The framing of our survey question, which specifically asked about changes “for the better and for the worse,” may have prompted caregivers to identify positive changes. Prior studies have found that positive psychological constructs, such as personal growth, optimism, and resilience, are associated with improved quality of life in pediatric and adult cancer patients [47-49]. Interventions that promote recognition of protective factors early in therapy may help caregivers maintain resilience, improve family functioning, and develop post-traumatic growth [50-53].

Some limitations must be considered in interpreting this study. Analysis was based on responses from one free-response question, which was optional in the context of a larger survey. Not all patients who were enrolled in the study completed this question, although demographics among children of respondents were similar to those of survey participants overall. Caregiver demographics were not collected, and it is possible that different family members completed the survey at different timepoints. Any comparison between timepoints must be interpreted with caution, as responses were not necessarily paired, with some caregivers only participating at earlier timepoints and some only at later timepoints. Among responses, there was also notable variability in the length and detail provided. Unlike an interview or focus group, we were not able to follow-up to clarify or expand upon responses. Nonetheless, the information gathered can be a helpful starting point in designing future surveys or more focused interview studies. In future analyses of caregiver experiences, it will also be important to identify caregiver demographics and whether experiences differ by demographic or socioeconomic groups.

In summary, caregivers identify a range of experiences during their child’s leukemia treatment. Negative changes were common and persistent across timepoints; however, many caregivers identified positive life changes, which may have new implications for targeted interventions to modify distress. The concerns voiced by families provide insight that can help healthcare providers offer targeted supportive care to enhance patient and family functioning during treatment.

## Supplementary Material

Supporting Information

Additional supporting information can be found online in the Supporting Information section.

**Supporting File**: pbc32057-sup-0001-SuppMat.docx

## Figures and Tables

**TABLE 1 ∣:** Participant characteristics.

	Respondents^a^ at ≥1 timepoint (*n* = 468)	Non-respondents^b^ (*n* = 87)	*p*-value
Age of child at enrolment,			
Age in years, median (SD)	5.6 (1.6)	5.7 (1.6)	0.66
	*n* (%)	*n* (%)	
**Sex of child**			0.24
Male	254 (54.3)	41 (47.1)	
Female	214 (45.7)	46 (52.9)	
**Race/Ethnicity of child**			0.79
Hispanic	76 (16.2)	13 (14.9)	
Non-Hispanic Asian	18 (3.9)	5 (5.7)	
Non-Hispanic Black	20 (4.3)	2 (2.3)	
Non-Hispanic White	324 (69.2)	63 (72.4)	
Other/Unknown	30 (6.4)	4 (4.6)	
**Marital status**			0.22
Married or living together	346 (73.9)	67 (77.0)	
Not married	91 (19.4)	11 (12.6)	
Unknown	31 (6.6)	9 (10.3)	
**Household income**			0.45
*<*$50,000	179 (38.3)	29 (33.3)	
≥$50,000	227 (48.5)	46 (52.9)	
Unknown	62 (13.3)	12 (13.8)	
**Insurance status**			0.70
Non-US	52 (11.1)	10 (11.5)	
US private	263 (56.2)	50 (57.5)	
US public	122 (26.1)	19 (21.8)	
Unknown/Self-Pay	31 (6.6)	8 (9.2)	

Abbreviation: SD, standard deviation.

a Caregivers who responded to the free-response question regarding family life at ≥1 timepoint.

b Caregivers who *did not* respond to the free-response question regarding family life at *any* timepoint.

**TABLE 2 ∣:** Number of caregivers completing overall survey and free-response question regarding family life at each timepoint.

Timepoint	Completed closed-ended survey	Responded to free-response question *n* (%)
T1	525	360 (68.6)
T2	352	219 (62.2)
T3	328	182 (55.5)
T4	314	169 (53.8)
T5	146	64 (43.8)

**TABLE 3 ∣:** Codes and subcategories identified in caregivers’ responses to free-response question on changes in family life.

Subcategory	Code	Explanation	Sample quote
**Negative changes**			
*Changes in daily routines*	*Missed activities*	Having to stop activities outside the home, canceled trips or events	“My son is unable to do swimming squad and piano lessons as he is too tired after school. We have limited our contact in crowded activities; it has been difficult to miss church and other activities in an effort to avoid germs” (R379T1)
	*Infection concern*	Concerns about patient’s risk of infections, changes to activities specifically to avoid exposure to sickness	“We can’t go do the simple things like go to a grocery store because we don’t want him to be exposed to any disease/germs. When my daughter got a cold we actually sent her to a friend’s house so [patient] would not get sick.” (R377T1)
	*Changes at school*	Missing school, concerns about school performance, switching to homeschooling	“Though he has had a homebound tutor, I am concerned about him starting school again and the impacts of the amount of time mis[s]ed.” (R17T2)
	*Childcare*	Need for more/different childcare for patient or siblings	“It has been a challenge juggling my child’s visits and getting care for my other child.” (R414T4)
	*Eating habits*	Change in eating habits	“The only the worse bad thing is [patient] don’t like eating what other are eating so we end up ordering out for him. That cost [sic] a lot of money.” (R392T4)
	*Life pace*	Change in pace of life, either difficulty keeping up with many demands, or feeling like things have slowed down	“We have slowed down and not moved forward with … goals that we had prior to dx.”/ “When (name of child) was first diagnosed we felt like we were being taken on a wild ride in an out-of-control vehicle with no option for a way out of it.” (R439T3)
	*Planning*	Difficulty with planning ahead	“Cannot plan anything as never know what day/night will bring” (R391T1)
*Work/Finances*	*Changes at work*	Change in employment or employment accommodations (e.g., work from home)	“Dad works extra hours. I quit my in home daycare and did not work through most of treatment; employer seems to be irritated regarding the length of FMLA” (R27T4)
	*Financial strain*	Added expenses, bills, income loss	“Household bills and needs are strained due to one income” (R43T2)
*Patient health and care needs*	*Patient symptoms*	Concerns about patient’s symptoms, including medical and behavioral	“Child’s moods are unpredictable and he does get tired of walking.” (R456T1) “I have to pay closer attention to her because of mood swings and fevers.” (R59T1) “We plan around her counts—we don’t always end up [doing] what we planned (i.e., when counts are low)” (R40T3)
	*Patient care needs*	Burden of multiple clinic appointments, hospitalizations, and daily medications	“Taking pills wearing masks coming here having pick [sic], spinal taps it’s all changed” (R372T1)
	*Travel for care*	Long commute to clinic or need to relocate for treatment	“Looking for a new vehicle with fewer miles due to so much travel to the hospital to appointments—live 2 h away from hospital.” (R453T2)
*Effects on other family members*	*Sibling stress*	Siblings are stressed, worried, or have to adjust (e.g., miss activities)	“Difficulty meeting siblings needs because of more frequent visits to clinic for the child who is the patient.” (R9T4) “Some resentment by other kids b/c M’s special treatment” (R12T1)
	*Relationship strain*	Fighting, strain to parents’ relationship, separation	“During our daughter’s intense chemo there was almost a double murder a few times. It is hard to comfort someone else (spouse) when you are in so much emotional pain yourself. During the time when our daughter was feeling very sick from the chemo it was like every man for himself.” (R279T2)
	*Parent health*	Parents’ health issues, parent fatigue, less time for parent self-care/sleep	“I have had literally no personal social life since the diagnosis of [child]’s ALL. I have been consumed with either probate or treatment and making time to deal with my medical issues.” (R210T5)
*Emotional changes*	*Stress/Worry (unspecified)*	Expressions of general stress, anxiety, sadness	“Now we live in a constant state of fear. It is hard to be happy about anything.” (R279T1)
	*Concern about the future*	Worry about relapse/what will happen in the future	“Lots more fear of unknown, what will happen down the road.” (R405T2)
	*Isolation*	Feeling lonely, lack of family or community support	“It’s been hard for me (mom) because we do not talk to anyone, we just stay at home.” (R330T1)
**Positive changes**			
*Family support*	*Family bonding*	Family feels closer, appreciate each other, more family time	“We become a strong family. We are more close as a family.” (R222T1)
	*Communication*	Improved communication, expressing feelings	“More communication with all family members. We have learned how we cope differently. We have learned how we process differently.” (R351T5)
*Community support*	*Community support*	Support from extended family, neighbors, school, etc.	“Have huge support system both sides of family; been very helpful with both pt and 10-year old brothers. Sons have been given much support from school community and athletic community we are a part of.” (R158T1)
	*Gifts*	Gifts and opportunities from organizations for cancer patients (e.g., make-a-wish), new friends as a result of cancer	“More opportunities for family activities due to organizations offering tickets to camps, events, etc.” (R9T2)
	*Giving back*	Empathy for other families, advocacy, raising awareness	“We’ve started a Foundation... and we try to help other families with financial situations and deliver Christmas trees to the kids spending Christmas in the hospital.” (R25T5)
*Changes in outlook*	*Re-evaluate priorities*	Change in values, realizing what is important	“Simple things like a good day out without any side effects are cherished more.” (R198T1)
	*Spirituality*	Finding strength in religion/faith	“Unexpected enlivining [sic] of our faith life and connection to our loving and benevolent God” (R12T4)
	*Improvement, compared with earlier in therapy*	Expressions of patient and family life being better now than earlier in treatment	“Apart from this he is enjoying school, and life as a whole is more normal since he has been on maintenance.” (R342T3)
	*Acceptance*	Coming to terms with diagnosis	“We have learned to adjust. We learn how to accept the things we can’t control and with prayers accept what is in store for us.” (R347T1)

**TABLE 4 ∣:** Proportion of respondents reporting codes at any timepoint.

	Number (%) of respondents reporting code^a,b^
Missed activities	154 (33)
Sibling stress	143 (31)
Financial strain	142 (30)
Patient symptoms	137 (29)
Changes at work	139 (30)
Stress/Worry	143 (31)
Infection concern	86 (18)
School changes	70 (15)
Relationship strain	61 (13)
Parent health	56 (12)
Life pace	50 (11)
Travel for care	45 (10)
Childcare	48 (10)
Eating habits	38 (8)
Isolation	35 (7)
Planning	36 (8)
Patient care needs	32 (7)
Concern about the future	17 (4)
At least one negative change	418 (89)
# Negative changes per respondent, median (range)	3 (0–11)
Family bonding	169 (36)
Community support	18 (4)
Re-evaluate priorities	101 (22)
Gifts	65 (14)
Improvement, compared with earlier in therapy	31 (7)
Spirituality	15 (3)
Giving back	41 (9)
Communication	26 (6)
Acceptance	37 (8)
At least one positive change	273 (58)
# Positive changes per respondent, median (range)	1 (0–6)

a For caregivers with responses at multiple timepoints, a theme was counted only once, even if repeated at a later timepoint. Overall, 468 caregivers responded at least at one timepoint.

b Themes within stressors and positive changes are not mutually exclusive, i.e., a caregiver could report more than one theme.

## Data Availability

The data that support the findings of this study are available on request from the corresponding author. The data are not publicly available due to privacy or ethical restrictions.

## Abbreviations:

ALL: acute lymphoblastic leukemia
COG: Children’s Oncology Group
NCI: National Cancer Institute
